# Augmenting an electronic Ising machine to effectively solve boolean satisfiability

**DOI:** 10.1038/s41598-023-49966-6

**Published:** 2023-12-21

**Authors:** Anshujit Sharma, Matthew Burns, Andrew Hahn, Michael Huang

**Affiliations:** Department of Electrical and Computer Engineering, University of Rochester, Rochester, NY 14627 USA

**Keywords:** Electrical and electronic engineering, Computer science

## Abstract

With the slowdown of improvement in conventional von Neumann systems, increasing attention is paid to novel paradigms such as Ising machines. They have very different approach to solving combinatorial optimization problems. Ising machines have shown great potential in solving binary optimization problems like MaxCut. In this paper, we present an analysis of these systems in boolean satisfiability (SAT) problems. We demonstrate that, in the case of 3-SAT, a basic architecture fails to produce meaningful acceleration, largely due to the relentless progress made in conventional SAT solvers. Nevertheless, careful analysis attributes part of the failure to the lack of two important components: cubic interactions and efficient randomization heuristics. To overcome these limitations, we add proper architectural support for cubic interaction on a state-of-the-art Ising machine. More importantly, we propose a novel semantic-aware annealing schedule that makes the search-space navigation much more efficient than existing annealing heuristics. Using numerical simulations, we show that such an “Augmented” Ising Machine for SAT is projected to outperform state-of-the-art software-based, GPU-based and conventional hardware SAT solvers by orders of magnitude.

## Introduction

Semiconductor fabrication technology and advanced von Neumann computer architecture have been two key drivers for the explosive increase in computing system performance in the past 50 years or so. As both drivers are reaching a phase of diminishing returns, interest in non-von Neumann systems is increasing. One obvious example is quantum computing (QC). QC has become more widely discussed ever since Peter Shor proposed an exponentially faster quantum algorithm for factorization^1^. However, we have yet to discover a wide array of applications where QC can change the complexity class of the solution algorithm. Grover’s algorithm is another popular quantum algorithm that theoretically provides a quadratic speedup for unstructured search^2^. In practice, however, realities of error sensitivity in the “Noisy-Intermediate Scale Quantum” (NISQ) era devices limit such algorithms’ immediate usefulness^3^. More noise-resilient, “shallow-depth” algorithms have been proposed^4–6^. So far, there is no indication that they can overcome NISQ era hurdles.

If we recall Feynman’s motivation of building quantum mechanical systems as computers^7^, we see that the argument applies to classical dynamical systems as well. Large-scale natural or artificial systems (e.g., weather or electronic circuits) can be modeled by ordinary or stochastic differential equations (ODE/SDE), but the modeling can be computationally very expensive. Yet nature seems to have little trouble “solving” these ODEs/SDEs effortlessly and often much more quickly than von Neumann systems. Hence building a *physical* dynamical system is in some sense building a “nature-based” computing system. One such type of dynamical systems that are gaining attention lately are often called *Ising machines*.

The Ising model is a physics-oriented model that describes the energy of a system of *N* coupled spins (See Methods section for more details). Finding the ground state of the Ising model is a classical example of combinatorial optimization problems (COP). Although early study focused on spins on 1D^8^ lattice, the prevailing notion of Ising model is not restricted to any graph topology, and is thus equivalent to the quadratic unconstrained binary optimization (QUBO) model. A generic QUBO problem is NP-complete and usually solved by physics-inspired Simulated Annealing (SA) algorithm. When an Ising machine maps such a problem, its dynamics can be extremely fast and much more efficient than SA on a von Neumann system. Quantum annealers (QA) marketed by D-Wave Systems^9^ are but early examples. The Coherent Ising machine (CIM) is another prominent example. CIM uses modulated optical pulses to represent spins^10^. In a recent work, NTT developed a 100,000-node CIM^11^, thus demonstrating a scalable implementation. Since the working principle of CIM can be attributed to the Kuramoto model^12^, researchers have proposed other electronic Oscillator-based Ising machines (OIM)^13^. Other emerging technologies have also been proposed to build Ising machines like memristors^14^ and p-bits^15^. A more thorough survey of Ising machines can be found in^16^.

In this paper, we will take an application-centric approach to analysing Ising machines with *boolean satisfiability (SAT)* as a case study^17^ (See Methods section for more details). SAT is an excellent benchmark: While many applications can be mapped into the Ising formula, there are nuances in the case of SAT problems: *high-degree interaction* and *efficient searching heuristic*. High-degree Ising machines have been proposed in the past^18–21^, but these completely ignored the second point. As we shall see, this has a serious implication on the machine’s capabilities to achieve state-of-the-art performance. For concreteness, our experiments will focus on an electronics-based CMOS-compatible Bistable Resistively-coupled Ising machine (BRIM)^22–25^. It has several key advantages compared to previous works. First, it uses the signs of the voltage across capacitors to represent Ising spins, thus making it easily realizable. Second, it uses programmable resistive coupling units, thus making it easy to implement all-to-all couplings. Third, it relies on natural dynamics of the system based on nano-scale capacitive spins, thus making it fundamentally more efficient than von Neumann accelerators.

The main contributions of this paper can be summarized as follows. First, we demonstrate that standard Ising machines are not competitive in solving SAT compared to state-of-the-art SAT solvers. Our analysis show that the main causes are lack of support for high-degree interaction and efficient search-space navigation. Second, motivated by these limitations, we extend a baseline Ising machine to support high-degree terms. Third, more importantly, we propose a new semantic-aware annealing heuristic to significantly improve Ising machines’ navigational efficiency. Finally, using numerical simulation, we demonstrate that our proposed “augmented” Ising machine is projected to be orders of magnitude faster than existing state-of-the-art SAT solvers.

## Results

### Solving 3-SAT using Ising machines

While Ising machines have been shown to be competitive in solving generic QUBO (general MaxCut) problems^22,23,26^, their application to SAT has not been as successful. This is partly due to the fundamental structure of *k*-SAT for $k\ge 3$: Given an *N*-variable, *M*-clause 3-SAT formula *F*, we can construct a minimization energy function (*H*) as follows:

1 $$\begin{aligned} \begin{aligned} F&= \bigwedge _{i=1}^M (\ell _{i1} \vee \ell _{i2} \vee \ell _{i3})\\ H&= \sum _{i=1}^M g(\ell _{i1})g(\ell _{i2})g(\ell _{i3}) \end{aligned} \end{aligned}$$

where,

2 $$\begin{aligned} \begin{aligned} g(\ell _{ij})&= {\left\{ \begin{array}{ll} (1-x_n) &{} \ell _{ij}=x_n\\ x_n &{} \ell _{ij}=\lnot {x}_n\\ \end{array}\right. } \end{aligned} \end{aligned}$$

where, $\ell _{ij}$ is the *j* *th* literal in the *i* *th* clause and $n\in \{1,2,\dots ,N\}$. Some state vector $x^*$ satisfies *F* if and only if $H(x^*)=0$. Here, $x^*$ is called the *ground state* of *H* (it is assumed that $x_n=1$ means *True* and $x_n=0$ means *False*). Any *unsatisfying* state *x* will have $H(x) > 0$.

It is not difficult to see that after expanding the summation, *H* will have cubic terms, $x_ix_jx_k$. However, the Ising/QUBO formula only supports upto quadratic (2nd-degree) terms. To get around this problem, the standard approach is to *quadratize* these high-degree terms^27–29^ to get a QUBO problem.

**Figure 1:**
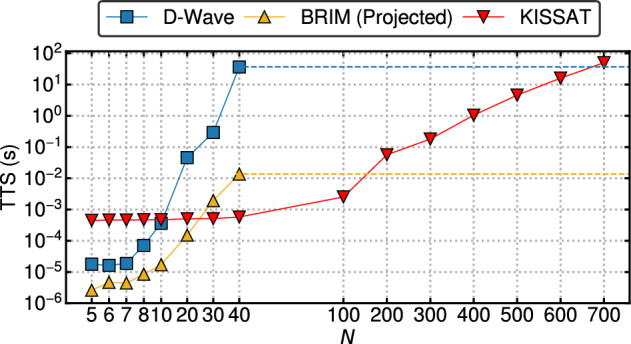
Time-To-Solution (TTS) of BRIM and D-Wave annealer compared against a state-of-the-art SAT solver, KISSAT^30^ for uniform random problems with varying number of variables, *N* and clauses, $M=4.25N$. The yellow and blue horizontal dashed line depicts how big of a problem KISSAT can solve for the same TTS taken by D-Wave and BRIM respectively while solving 40-variable problems.

Figure 1 shows the Time-To-Solution (TTS) of BRIM and D-Wave’s QA after quadratization, compared against a state-of-the-art SAT solver, KISSAT^30^. TTS^31^ is defined as the expected time to find a solution with 99% probability. Over multiple instances each with runtime of $T_{run}$ and a success probability of *P*, TTS can be calculated using the following formula:

3 $$\begin{aligned} TTS = {\left\{ \begin{array}{ll} T_{run}\times \dfrac{\log _{10}(0.01)}{\log _{10}(1-P)} &{} P < 0.99\\ T_{run} &{} \text {otherwise} \end{array}\right. } \end{aligned}$$

For SAT, success probability *P* is the proportion of runs that found satisfying states out of all the runs for a given problem. Each data point in the figure is the geometric mean TTS of 10 different problem instances. We can observe that while BRIM’s trend is much better than that of D-Wave, compared to KISSAT, the initial speed advantage of both the Ising machines is quickly lost with only a small increase in problem size. The horizontal dashed lines show that for the same TTS that D-Wave and BRIM takes to solve a 40-variable problem, KISSAT can solve $\sim$700 and $\sim$150-variable problems respectively. There are two reasons for this disappointing performance of Ising machines:

**Figure 2:**
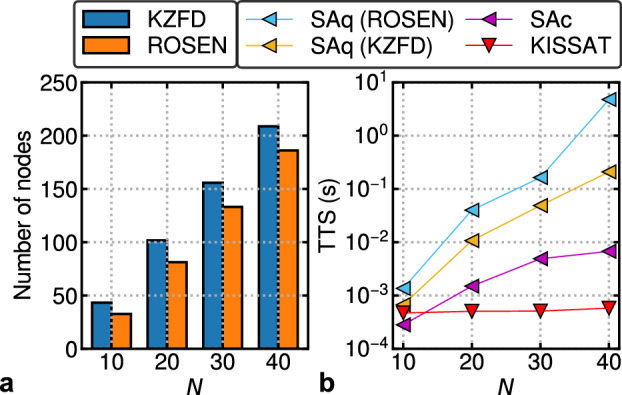
Illustration of drawbacks of Ising machines. (**a**) The average number of nodes after quadratization for increasing number of variables. (**b**) Performance of SA on the QUBO formulations (SAq) and on the original hypergraph with support for cubic interaction (SAc) compared against KISSAT.

1. *Lack of support for high-degree interactions*: Fig. 2a shows the resultant number of nodes after using Kolmogorov-Zabin-Freedman-Drineas (KZFD)^28,29^ and Rosenberg^27^ quadratization for increasing number of variables. We can observe that roughly, there is a 4-5x increase in the number of nodes compared to the number of variables, resulting in an increased number of phase points from $2^N$ to $\sim 2^{5N}$. This is because both quadratization techniques introduce roughly 1 extra variable per clause. Therefore, the resultant number of nodes is $\sim (N+M)$, where $M\approx 4.25N$ for hard uniform random 3-SAT instances. Such a massive disadvantage has been demonstrated in earlier works which motivated many researchers to propose Ising machines with high-degree interactions^18–21^. Figure 2b shows the TTS of SA^32^ on the QUBO formulations (SAq) and with cubic interaction support (SAc) compared against KISSAT. We can observe that SAc significantly outperforms SAq, thus showing the importance of high-degree interactions. However, it still scales poorly when compared to KISSAT, which brings us to the next point.
2. *Lack of efficient searching heuristics*: After converting to the Ising/QUBO formulation, insights from the original problem formulation may not be obvious. However, such insights are exploited by software SAT solvers like KISSAT to effectively search the landscape^33^. This gap between SAc and KISSAT is the result of efficient searching heuristics. Previous works on high-degree Ising machines completely ignored the vast amount of insights from SAT competitions^34^ and literature^33,35,36^.

These two shortcomings motivate us to propose hardware support for cubic interaction and a semantic-aware heuristic to effectively guide the Ising machine towards a solution as we will discuss in the next section.

Apart from these limitations, D-Wave QA also suffers from an additional drawback: lack of support for all-to-all coupling. The limited connectivity of the hardware topology necessitates a costly graph embedding process (which is itself NP-hard) that greatly increases the final graph size^37^. This is the major reason for its worse performance scaling compared to BRIM (which supports all-to-all couplings) in Fig. 1. Any Ising machine only supporting near-neighbor couplings will suffer from this fundamental limitation^22^.

### BRIM with support for cubic interaction

Let us rewrite the 3-SAT objective function (Eq. 1) by collecting linear, quadratic, and cubic terms:

4 $$\begin{aligned} \begin{aligned} H = const - \sum _n l_nx_n - \sum _{n<j} q_{nj}x_nx_j - \sum _{n<j<k} c_{njk}x_nx_jx_k \end{aligned} \end{aligned}$$

In BRIM, each variable $x_n$ is represented by a continuous capacitive voltage $v_n\in [0,1]$. Moreover, the output nodal voltage is *quantized* to {0,1} V with 0.5 V being the threshold. A real quantizer can be represented as a scaled and shifted-tanh function $Q(v_n)$ with a steep slope, $S>0$. Hence, we get a continuous variable energy function $\tilde{H}$ from Eq. (4):

5 $$ \begin{aligned}&\tilde{H} = const - \sum _n l_nQ(v_n) - \sum _{n<j} q_{nj}Q(v_n)Q(v_j) - \sum _{n<j<k} c_{njk}Q(v_n)Q(v_j)Q(v_k)\\&\text {where, }Q(v_n) = 0.5\cdot tanh\{S\cdot (v_n-0.5)\} + 0.5;\, x_n = \lim _{S\rightarrow \infty } Q(v_n) \end{aligned} $$

Now, the incoming current for each node is given the following equation:

6 $$\begin{aligned} \begin{aligned} \boxed {\frac{dv_n}{dt} = \alpha \Big (l_n + \sum _{j} q_{nj}Q(v_j) + \sum _{j,k} c_{njk}Q(v_j)Q(v_k)\Big ) = -\alpha \frac{\partial \tilde{H}}{\partial Q(v_n)} = -\frac{\alpha }{0.5\cdot S\cdot \mathrm sech^2\{S\cdot (v_n-0.5)\}}\cdot \frac{\partial \tilde{H}}{\partial v_n};\,\alpha > 0} \end{aligned} \end{aligned}$$

**Figure 3:**
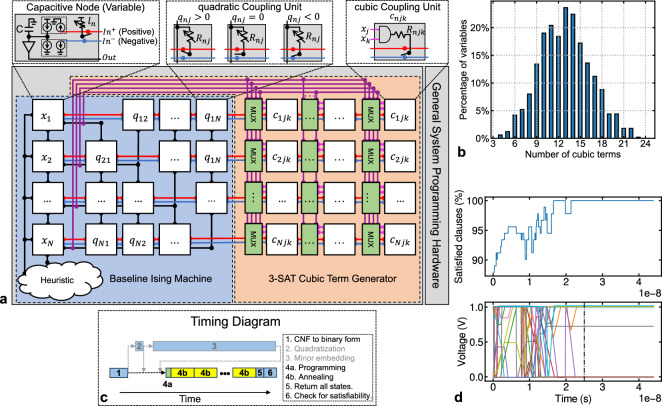
Illustration of cBRIM. (**a**) High-level cBRIM diagram extending the baseline BRIM with MUXs and cubic coupling units. (**b**) Distribution of number of variables participating in cubic terms. (**c**) Timing diagram of using cBRIM. The greyed-out steps are not required for cBRIM (but may be required for other Ising machines). (**d**) The evolution of the number of satisfied clauses (%) [top] and nodal voltages for all nodes in cBRIM [bottom] while solving a 20-variable problem. The vertical dash line indicates that a solution is found when the voltages have settled.

We modify the baseline BRIM as proposed by Afoakwa et al.^22^ for bit-{0,1} representation as shown in the blue block in Fig. 3a. This supports the first two terms ($l_n + \sum _{j} q_{nj}Q(v_j)$) in the parenthesis of Eq. (6). There are *N* bi-stable capacitive nodes whose voltages range $\in [0,1]$. The bit value of each node is simply the *quantized* voltage $\in \{0,1\}$. Thus, each node represents a variable in the SAT formula. Every node is connected to its neighbors via programmable resistive coupling units *q*. This resistance between node *n* and node *j* is given by $R_{nj} = \frac{R}{|q_{nj}|}$, where *R* is a constant resistance and $q_{nj}$ is normalized to $[-1, +1]$. Thus, strong coupling means lower resistance. The sign of $q_{nj}$ is implemented as follows: when $q_{nj} > 0$, the nodes are coupled in a parallel fashion (output of one node is connected to the positive input terminal of the other, via resistance $R_{nj}$: $Out_n\rightarrow In^+_j,\ Out_j\rightarrow In^+_n$); when $q_{nj} < 0$, they are coupled in an anti-parallel fashion ($Out_n\rightarrow In^-_j,\ Out_j\rightarrow In^-_n$); nodes are disconnected if $q_{nj} = 0$. Each node *n* can also have a linear bias $l_n$.

To support the last term ($\sum _{j,k} c_{njk}Q(v_j)Q(v_k)$), we require 3 steps: ${}^{\textcircled {1}}$ use a multiplier to produce a voltage $v_{jk} = Q(v_j)\times Q(v_k)$, ${}^{\textcircled {2}}$ apply $v_{jk}$ across a resistor proportional to $\frac{1}{c_{njk}}$, and ${}^{\textcircled {3}}$ feed the current to node *n*. In practice, we make 3 optimizations as we discuss next:

1. *Multiplier:* A “true” analog multiplier is a rather expensive circuit due to accuracy and linearity concerns. Since the differential equation (Eq. 6) itself is a continuous approximation of discrete variables and the output voltage of nodes is *quantized*, we use a simple AND gate as a multiplier.
2. *Cubic terms fan-in:* For a *general* support of cubic interaction, we need $O(N^3)$ couplers. However, for 3-SAT, the number of cubic terms is determined by the number of clauses, *M* which, for difficult uniform random problems, is typically about 4.2*N*^38^. Therefore, *on average*, each variable is involved in about a dozen cubic terms—regardless of the size of the problem. Figure 3b shows the distribution of variables occurring in cubic terms for uniform random problems. As we can observe, the plot doesn’t have a long tail and a fixed degree fan-in of $d=20$ would work. Every multiplier (AND gate) is fed by two *N*:1 multiplexers to program which voltages to multiply. This keeps the hardware complexity at $O(N^2)$. Given a fan-in limit chosen for a particular system, users can pre-process excess clauses with quadratization techniques.
3. *Coupler programmability:* Finally, another problem-specific feature of 3-SAT is that the vast majority of cubic term coefficients are $\pm 1$. For an *M*-clause 3-SAT problem, there are at most *M* cubic terms produced by Eq. (1), one for each clause with 3 literals. On rare occasions, when the magnitude of a coefficient is greater than 1, that cubic term is simply treated as multiple instances that take up multiple couplers (fan-in). So we eliminate the programmability on the coupler strength—it will be fixed at 1. Instead, the programmability is on which two inputs to multiply, and the polarity of the coupling.

The resulting architecture is shown in Fig. 3a. We name this design, cubic BRIM or cBRIM in short. By minimizing $\tilde{H}$, cBRIM maximises the number of satisfied clauses and thus, solves Max-3-SAT. When there is no random perturbation, using Eq. (5) and Eq. (6), we can show the Lyapunov stability analysis as follows:

7 $$\begin{aligned} \begin{aligned} \boxed {\frac{d\tilde{H}}{dt} = \sum _n\frac{\partial \tilde{H}}{\partial v_n}\cdot \frac{dv_n}{dt} = \sum _n\frac{\partial \tilde{H}}{\partial Q(v_n)}\cdot \frac{\partial Q(v_n)}{\partial v_n}\cdot \frac{dv_n}{dt} = -\frac{1}{\alpha }\sum _n\Big (\frac{dv_n}{dt}\Big )^2\cdot \frac{\partial Q(v_n)}{\partial v_n}=-\frac{1}{\alpha }\sum _n\Big (\frac{dv_n}{dt}\Big )^2\cdot 0.5\cdot S\cdot sech^2\{S\cdot (v_n-0.5)\}} \end{aligned} \end{aligned}$$

Now, $v^*$ is a solution to a 3-SAT problem if and only if $\tilde{H}(v^*) = 0$. Otherwise, $\tilde{H} > 0$. Moreover, since $\alpha , S > 0$ and $v_n\in [0,1]$, from Eq. (7), $(\frac{d \tilde{H}}{dt} \le 0)$ for all $v \ne v^*$. Therefore, the system is Lyapunov stable: it naturally seeks local minima of $\tilde{H}$ and stays there when found. Figure 3d shows the evolution of the number of satisfied clauses (%) [top] and nodal voltages in cBRIM [bottom] while solving a 20-variable problem. The voltages settle at about 25 ns and the quantized voltages indeed satisfies the 3-SAT formula.

Figure 3c shows the timing diagram of using cBRIM. All the blue boxes represent von Neumann computation. Steps 2 and 3 are not required for cBRIM and hence, greyed out. In step 1, the given 3-SAT problem in Conjunctive Normal Form (CNF) is pre-processed via $O(3d_{max}\cdot M + N)$ time von Neumann computation (where $d_{max}$ is the maximum degree of the graph) to turn it into a binary formulation (Eq. 4). The formulation is organized as a matrix, and programmed into the Ising machine hardware column by column in step 4a. For linear and quadratic terms, the programmed values represent the coefficient of terms. For cubic terms, it identifies the variables to be multiplied together. The Ising machine is then annealed multiple times, each time storing the final state (step 4b). After the annealing process, the system returns all the binary state vectors to the von Neumann computer (step 5) which then checks each state for satisfiability (step 6). The Ising machine can also store snapshots of the spin vector in a run which can be used to get the evolution of number of satisfied clauses.

### Semantic-aware randomization

A simple yet effective annealing schedule is to randomly flip a spin with a certain probability and gradually reduce this probability^22^. Such a problem-agnostic heuristic works well to escape local minima for problems that lack any structure. However, SAT is a well-studied problem whose structure is exploited by solver heuristics. Conflict Driven Clause Learning (CDCL) solvers learn new clauses using Boolean resolution to actively store information on the problem at hand, and Variable State Independent Decaying Sum (VSID) has been shown to exploit large scale problem structures^39^. Stochastic Local Search (SLS) algorithms such as WalkSAT^40^ include a veritable buffet of different heuristics for variable selection based on various scoring and ranking mechanisms. With this motivation, we propose a new heuristic.

#### Tanh-make-break (TMB) heuristic

 The *tanh-make-break* (TMB) heuristic assigns probability, $p_n$ to flip each variable $x_n$ as follows:

8 $$\begin{aligned} \begin{aligned} p_n = \tanh (c_m\times \mathscr {M}_n)\cdot [1-\tanh (c_b\times \mathscr {B}_n)] \end{aligned} \end{aligned}$$

where, $c_m > 0$ and $c_b > 0$ are parameters. Given an assignment of variables $\texttt{a}$, $\mathscr {M}_n$ represents the number of newly satisfied clauses after flipping a variable $x_n$ (*make count*), while newly unsatisfied clauses contribute to $\mathscr {B}_n$ (*break count*). Our main motivation to use the $\tanh$ function are: First, it is easily implementable in hardware. Second, it directly resembles probability without any need for normalization. Third, it is non-linear as experimentally we found it to work better. Finding the most optimal function is left as future work. The main intuition for TMB is that, stochastically flipping nodes that satisfy more clauses makes the landscape navigation more efficient as we shall see later. An illustrative example is shown in the supplemental. A key point to note here is that, unlike SLS algorithms, TMB allows multiple nodes/variables to flip in parallel without any synchronization. This makes the hardware design much simpler.

To compute $p_n$, we need to compute $\mathscr {M}_n$ and $\mathscr {B}_n$. It turns out, the incoming electric current for each node in our system is related to its $\mathscr {M}_n$ and $\mathscr {B}_n$ as follows:

9 $$\begin{aligned} \begin{aligned} \frac{dv_n}{dt}&= \alpha (1-2x_n)\cdot (\mathscr {M}_n-\mathscr {B}_n) \end{aligned} \end{aligned}$$

The derivation is shown in the Supplemental material. With this relation, we opt to compute $\mathscr {M}_n$ since it is much easier to obtain (refer to the supplemental for more details) and recover $\mathscr {B}_n$ from the incoming electric current in Eq. (9).

**Figure 4:**
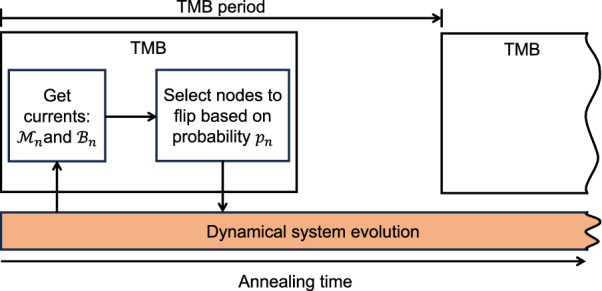
The high-level flow of how TMB works with our dynamical system.

Figure 4 shows how we integrate TMB into our dynamical system. For each node, after every *TMB period* time, the heuristic gets the electric currents proportional to $\mathscr {M}_n$ and $\mathscr {B}_n$. This information is used to select the respective node to flip determined by the probability $p_n$ as in Eq. (8). The selected nodes are then temporarily connected to $V_{DD}$/ground to charge/discharge the capacitor respectively. Note that, while TMB is in the works, the system dynamics keeps evolving. If the system found a satisfying solution before the cutoff time, then $\forall n\,(\mathscr {M}_n=0)$. In this case, the system stops annealing and returns the spin vector (*latching*).

### High-level analyses of Augmented Ising Machine for SAT (AIMS)

**Figure 5:**
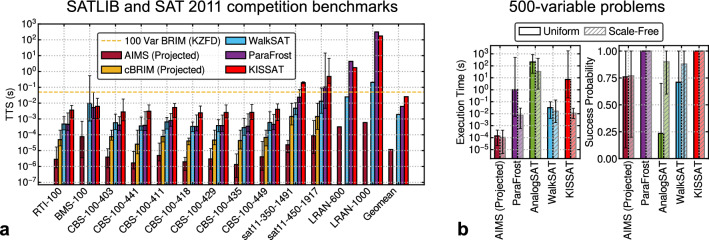
Performance comparison of AIMS against existing SAT solvers. (**a**) Comparing TTS of various solvers for benchmark suites from SATLIB^41^ and SAT 2011 competition. Each benchmark suite is named in this format: $\langle$Benchmark$\rangle$–$\langle$Vars$\rangle$–$\langle$Clauses (if stated)$\rangle$. (**b**) Plots showing the execution time [Left] and success probability [Right] for various solvers. All solvers run 20 problems each with uniform random and industrial-like scale-free distribution of 500 variables.

With the discussed architectural innovations: cubic interaction and TMB heuristic, we propose Augmented Ising Machine for SAT (AIMS). We tuned the parameters $c_m$ and $c_b$ in our TMB heuristic that works the best, in general, for all problems. Sensitivity analysis is discussed in the supplemental. All the results are obtained using numerical simulations with realistic device parameters, assuming a 45nm technology. AIMS is first compared against state-of-the-art software solvers and cBRIM. Note that, existing high-degree Ising machines have been either evaluated with tiny problems^19^ or with poor solution quality and no direct report of the solver time^18^. In contrast, cBRIM is able to find solutions with higher success rates and hence, is compared here. Figure 5a shows the geometric mean TTS for each benchmark suite with error bars and also the overall TTS for various solvers.

As we can observe, for these benchmarks, AIMS is consistently faster with a geometric mean speedup of about 2 and 3 orders of magnitude over software solvers WalkSAT^40^ and KISSAT^30^ respectively. The performance of the GPU-based SAT solver, ParaFrost^42^ lies between WalkSAT and KISSAT, thus it is slower than AIMS by about 517x. With contributions from our TMB heuristic, AIMS outperforms cBRIM by at least an order of magnitude. Some of the data for cBRIM are missing as it could not solve the problems in our tested annealing times. The horizontal dashed line shows the TTS of baseline BRIM solving the QUBO formulation of a 100-variable problem. The speed of AIMS compared to both cBRIM and BRIM emphasizes the importance of our proposed architectural innovations.

Now, we look at the execution times and success probabilities for various solvers on 500-variable uniform random and scale-free problems as shown in Fig. 5b. The results of WalkSAT and KISSAT are shown for reference. AnalogSAT ^43^ is a recently proposed GPU-based solver that accelerates a simulation of a dynamical system. We can observe that AIMS takes the least execution times of $\sim 100$ μs on average while still maintaining high success probability of 0.75. For both the problem types, AnalogSAT performed the worst as it took the longest times with low success probability for uniform random problems. KISSAT slightly outperformed WalkSAT in scale-free problems which corroborates its success in industrial SAT instances. In terms of TTS, AIMS is faster than the better performing GPU-based solver, ParaFrost, by about 3 orders of magnitude for uniform random problems and by 27$\times$ for scale-free problems.

**Table 1:** Comparison of TTS for various hardware solvers in units of µs.

Benchmark	*N*	*M*	HW AmoebaSAT^44^	BRWSAT^45^	AIMS (projected)
uf50-0100	50	218	4.126	−	5.829
uf50-0410	50	218	4.360	−	1.329
uf50-0767	50	218	8.300	−	4.059
uf100-0285	100	430	356	−	73.81
uf150-0100	150	645	1832	−	73.16
uf225-028	225	960	3078	−	10.41
sat11-350-p11	350	1491	−	200	39.54
sat11-350-p23	350	1491	−	1289	44.56
sat11-350-p75	350	1491	−	280	22.81
sat11-350-p98	350	1491	−	50	7.389
sat11-450-p1	450	1917	−	80	6.448
sat11-450-p11	450	1917	−	1912	577.1
sat11-450-p14	450	1917	−	4650	786.2
sat11-450-p78	450	1917	−	353.1	8.082

Apart from software solvers, we also compare AIMS with two other state-of-the-art hardware SAT solvers: FPGA-based HW AmoebaSAT^44^ and Magnetic Tunnel Junction (MTJ)-based BRWSAT^45^ as shown in Table 1. The benchmarks shown here are the only ones reported by the references and hence, can be directly compared. Based only on the overlapping benchmarks, AIMS has a geometric speedup of 7.4$\times$ and 10.3$\times$ over HW AmoebaSAT and BRWSAT, respectively. Note that HW AmoebaSAT scales rather poorly as TTS increases 3 orders from problem sizes of 50 variables to 225 variables.

Finally, using Cadence simulations, we estimate that a 500-variable AIMS chip will consume $\sim 300$ mW power and require $\sim 13\times 13\,{\text {mm}}^2$ area. A detailed table of hardware parameters is shown in the supplemental. We believe that there is room for significant improvement for the various circuits used in this study.

### Navigational efficiency

**Figure 6:**
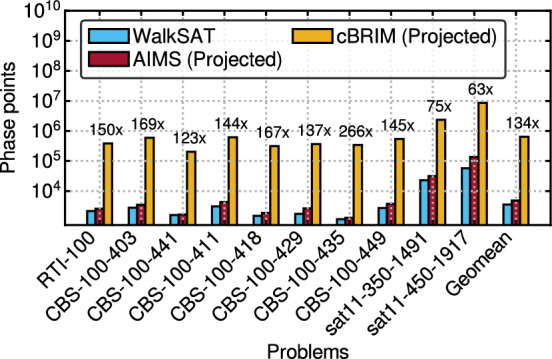
Geometric mean phase points visited by AIMS, cBRIM and WalkSAT for various benchmark suites from SATLIB and 2011 SAT competition. The labels show the improvement of AIMS over cBRIM.

With such promising results demonstrated in the previous section, it is important to also look at how efficiently AIMS finds a solution. Figure 6 shows the geometric mean phase points visited to arrive at a solution by AIMS, cBRIM and WalkSAT. A phase point denotes a specific state of the quantized spin vector $\in \{0,1\}^N$. Each such state traversed by AIMS in a run is counted as a phase point “visit”. Since KISSAT is a CDCL solver, the notion of a phase point is not well defined and hence is not shown. Note here that the number of phase points visited is also adjusted by solution probability as in TTS. AIMS is consistently more efficient than cBRIM as it could achieve a satisfying solution by visiting about 134$\times$ fewer phase points in general. Note that some of the benchmarks are not shown since cBRIM fails to find a solution for these, suggesting that it may require searching through many more phase points. WalkSAT still requires slightly fewer phase point visits than AIMS in general. However, the extremely fast dynamics of AIMS more than compensates for the larger phase point visits with an average flip-rate of 4250 flips/µs compared to 6 flips/µs for WalkSAT. This explains the observed speedup of AIMS over WalkSAT in Fig. 5.

## Discussion

With diminishing speed of improvements for conventional computing systems, non-von Neumann systems such as Ising machines are receiving increased attention. While all COPs can be targeted by an Ising machine in theory, we have shown in this paper, using SAT as a case study, that there are still practical hurdles with such systems: high-degree interactions and efficient navigational heuristics.

Fortunately, these issues can be overcome with additional architectural support, at least for electronic Ising machines such as BRIM^22^. We have presented the design details to support cubic interaction natively in BRIM with 3 optimizations: ${}{\textcircled {1}}$ using AND gate as a multiplier, ${}{\textcircled {2}}$ exploiting SAT structure to still use $O(N^2)$ couplers and ${}{\textcircled {3}}$ using fixed resistors for the cubic couplers without much loss of generality.

More importantly, we have proposed a novel, semantic-aware annealing heuristic called TMB. To efficiently incorporate TMB into our dynamical system, we showed a relation between the difference $(\mathscr {M}_n-\mathscr {B}_n)$ for each variable and its total incoming current. Then we derived an expression to easily compute $\mathscr {M}_n$ using analog circuits and extract $\mathscr {B}_n$ from the above mentioned relation. This reduced the extra circuit complexity and made the design more feasible. With the proposed architectural support, the resulting augmented Ising machine for SAT (AIMS) can achieve orders of magnitude speedup over state-of-the-art SAT solvers. Moreover, we also demonstrated that AIMS can find a solution by visiting significantly fewer phase points than cBRIM, thus emphasizing the importance of TMB.

Although we conducted some non-ideal simulations verified partly with Cadence, a real hardware-based study is required in the future. Overall, our study suggests that Ising machines offer a powerful substrate to explore novel non-von Neumann computing. At the same time, additional architectural support may be crucial to truly unlock their performance potential.

## Methods

### Ising model

The Ising model was originally used to describe the Hamiltonian (*H*) for a state of spins ($\sigma _i$) on some lattice. A modified version (Eq. 10) of the formula is more useful in the discussion of optimization problems.

10 $$\begin{aligned} \begin{aligned} H(\sigma )&= -\sum _{(i,j)}J_{ij}\sigma _i\sigma _j - \sum _{i}h_i\sigma _i = -\sigma ^{\top }J\sigma -h^\top \sigma \end{aligned} \end{aligned}$$

It turns out, this optimization problem is NP-complete and all the Karp’s original set of NP-complete problems have been transformed into the Ising model^46^. A loose physical analogy is as follows. A system of spins ($\sigma _i \in \{-1, +1\}$) are subject to the influence of a set of parameters $J_{ij}$ and $h_i$. $J_{ij}$ describes the coupling between two spins ($\sigma _i$ and $\sigma _j$), while $h_i$ describes the influence of an external magnetic field on spin $\sigma _i$. Given this setup, the spins will naturally gravitate towards the lowest energy state (ground state). Technically, the probability of each state follows the Boltzmann distribution:

11 $$\begin{aligned} P(\sigma ) \propto e^{-\frac{H(\sigma )}{T}} \end{aligned}$$

### Boolean satisfiability (SAT)

A generic SAT problem is about determining whether a propositional logical formula *F* is satisfiable for some mapping of variables to truth values^17^. *F* consists of boolean-valued variables acted on by unary logical operator (*negation*: $\lnot$) or binary ones (*and*: $\wedge$; *or*: $\vee$; and *implications*: $\rightarrow$, $\iff$). For example, $F=x_1\vee (\lnot x_2)\wedge (x_3\rightarrow x_4)$ would be a formula in propositional logic. The statement is typically expressed in Conjunctive Normal Form (CNF)^17^, i.e. a logical conjunction of separate *clauses* (e.g. $F=C_1\wedge C_2 \wedge C_3$). Each clause is a logical disjunction of a number of *literals*. Literals are boolean variables or their negations (e.g. $C=x_1 \vee \lnot x_2 \vee x_3$). Any formula in propositional logic can be converted to CNF in linear time by introducing new variables and clauses^47^. When the number of literals in any clause is no more than *k*, the problem is called *k*-SAT. When $k\ge 3$, the problem is NP-complete. 3-SAT can be used to formulate any NP-complete problem in standard CNF, with SAT solvers as an efficient means of solution^48^.

### D-Wave QA results

We report the results on D-Wave’s 2000Q system with optimized annealing times of $2$ µs, $20$ µs and $2000$ µs for 5–7, 8–20, and 30–40 variable problems respectively. The results of the newer D-Wave Advantage system (5000 qubits) were worse for our experiments although it could map larger 3-SAT problems. This is consistent with previous studies which conjectured that for sparse problems like SAT, the large number of unused couplers in the *Pegasus* topology of D-Wave Advantage might lead to more noise^49,50^.

### Software-based solver results

In all the experiments, CPU software solvers like WalkSAT^40^, KISSAT^30^ and SA^32^ are natively executed on Intel Xeon Platinum 8268 CPU at 2.90 GHz. WalkSAT is run using the -best heuristic and cutoff flips of 500,000 with 20 retries. KISSAT is allowed to run with no cutoff time. GPU-based solvers (ParaFrost^42^ and AnalogSAT^43^) are executed on a system with AMD Ryzen Threadripper PRO 3975WX CPU with 16 GB of 3200 MHz DDR4 RAM and an NVIDIA GeForce RTX 3090 @ 1.70GHz GPU.

### Simulation of dynamical systems

The evolution of various dynamical systems like BRIM, cBRIM and AIMS is modeled by solving differential equations using 4th-order Runge-Kutta method in C++. The behavioral simulation is verified to be accurate upto 32 nodes when compared against detailed circuit simulations in Cadence.

### Benchmarks

For SAT benchmarks, we use those publicly available from SATLIB^41^ and the 2011 SAT competition. Moreover, we generated “hard” uniform random instances of varying sizes with $M/N = 4.25$. Since it has been established that industrial problems exhibit *scale-free* behaviour^51^, we generated some difficult industrial-like SAT instances with 500-variables using scale-free distribution with $M/N=3.29$ and power-law exponent of 2.935^51^. All problems are verified as satisfiable.

### Supplementary Information

Supplementary Information.

## Data Availability

The data that support the findings of this study are available from the corresponding author upon reasonable request.
