# Supplementary material for: Augmenting an electronic Ising machine to effectively solve boolean satisfiability
Source: Sci Rep. 2023 Dec 21;13:22858. doi: 10.1038/s41598-023-49966-6 (PMC10739962; doi:10.1038/s41598-023-49966-6)
Supplement: Supplementary file 1 — Supplementary Information. [file 41598_2023_49966_MOESM1_ESM.pdf]

Augmenting an Electronic Ising machine to effectively solve Boolean Satisfiability

Anshujit Sharma[1,*], **Matthew Burns**[1], **Andrew Hahn**[1], **and Michael Huang**[1]

[1]Department of Electrical and Computer Engineering, University of Rochester, NY 14627, USA
[*]ashar36@ur.rochester.edu

ABSTRACT

Supplementary Notes

Circuit for BRIM and AIMS

A circuit-based implementation has been developed in Cadence and simulated with the Virtuoso analog design environment. The design of the coupling units and nodes is shown in Fig. S1 and Fig. S2, respectively.

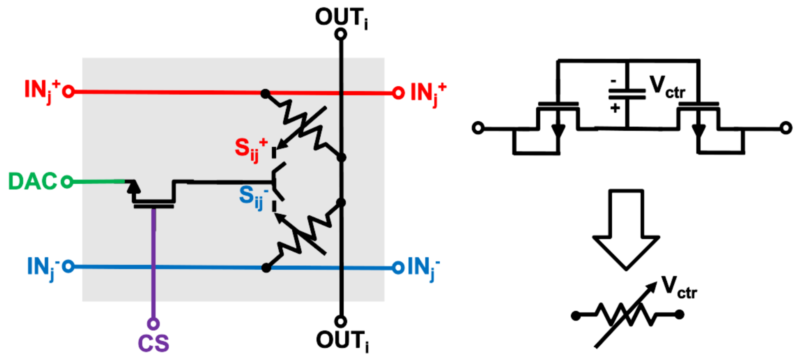

Supplementary Figure S1. [Left] Architecture of a coupling unit in hardware. [Right] Design of the variable resistor used to program the coupling weights.

The coupling unit includes two programmable resistors and a pair of switches. To enable positive coupling, S_{ij}^{+} is closed, connecting OUT_i to IN_j^{+} through a programmable resistor. To enable negative coupling, S_{ij}^{-} is closed, which connects OUT_i to IN_j^{-}. S_{ij}^{+} and S_{ij}^{-} are mutually exclusive, only one can be closed at a time for the circuit to operate as intended.

Implementing resistors directly on-chip is costly in terms of area and does not allow for programmable values to account for different coupling strengths between nodes. To circumvent both of these issues, rather than using a physical resistor, two p-type transistors are connected with a capacitor across the source and gate as shown in Fig. S1 [Right]. This configuration has a similar I-V characteristic to a physical resistor while consuming less area. By setting the voltage (V_{ctr}) across the capacitor, the effective resistance can be tuned, allowing for programmable resistances.

The node design in Fig. S2 includes a capacitor, which stores the current state of the node, and a buffer to quantize the output to either V_{SS} or V_{DD}. The node takes two inputs, i_{ref}^{+} and i_{ref}^{-}, which are currents due to positive and negative couplings respectively. The current i_{ref}^{+} is mirrored as i^{+} flowing from V_{DD} to the positive plate of the capacitor, thus charging it. Whereas, i_{ref}^{-} is mirrored as i^{-} such that it flows out of the positive plate to ground, thus discharging the capacitor. Therefore, the net current flowing into the positive plate of the capacitor is $(i^{+} - i^{-})$. With this arrangement, more positive couplings would result in charging the capacitor while the capacitor will discharge if there are more negative couplings.

We tested the Cadence implementation with a fully-connected 6-node MaxCut graph using all possible initial states. The system settled into a local minimum for every possible input, with an average power draw of 200 μW. Preliminary analysis revealed that the power consumption scales with the number of active coupling units and the number of nodes at V_{DD}. Taking into account the cubic couplings, couplings to generate $\mathscr{M}_n$ and TMB hardware in AIMS, a 500-variable SAT problem is

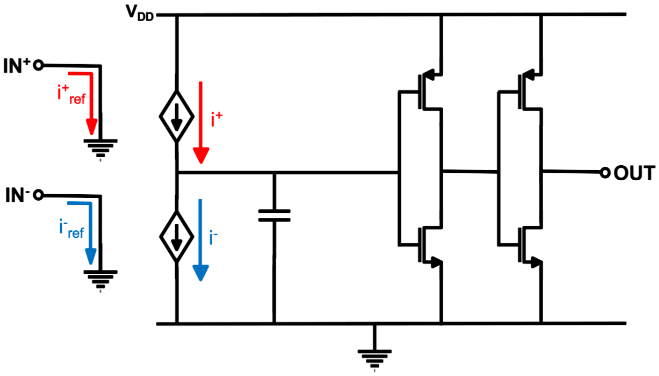

Supplementary Figure S2. Circuit architecture of a node. The node consists of two current mirrors (left), a capacitor, and a buffer to quantize the voltage (right).

estimated to consume $\sim 300\,mW$ power and require $\sim 13 \times 13\,mm^2$ chip area.

Illustrative example of $\mathscr{M}_n$, $\mathscr{B}_n$ and TMB

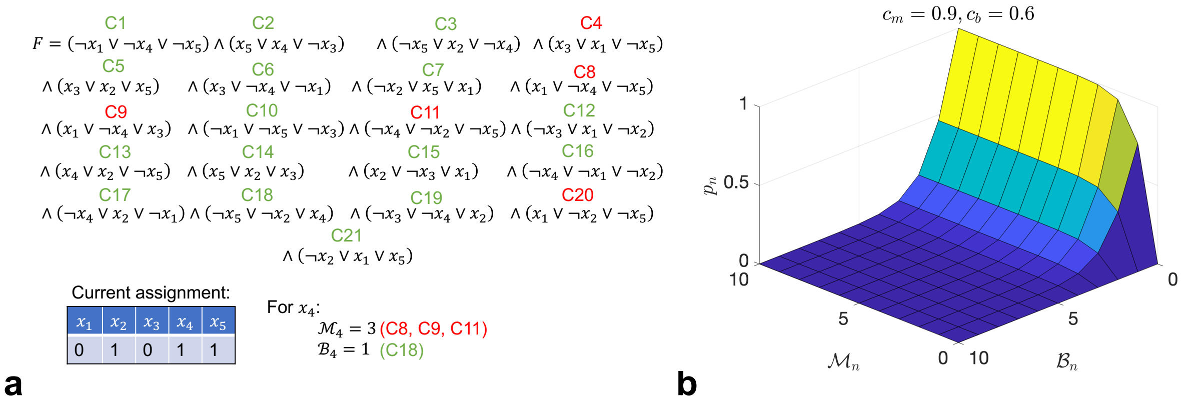

Supplementary Figure S3. Visualization of TMB. a) Example illustration of *make count* ($\mathscr{M}_n$) and *break count* ($\mathscr{B}_n$) for the variable x_4. Clauses are numbered C1 to C21 with *green* indicating satisfied and *red* indicating unsatisfied with the current assignment. b) 3D Plot of p_n as a function of $\mathscr{M}_n$ and $\mathscr{B}_n$ for a tuned c_m and c_b value.

Fig. S3a shows an example illustration of $\mathscr{M}_n$ and $\mathscr{B}_n$ for the variable x_4 in formula F. With the shown current assignment, if variable x_4 is selected to be flipped, it will satisfy 3 new clauses ($\mathscr{M}_4 = 3$) C8, C9 and C11, however, 1 new clause ($\mathscr{B}_4 = 1$) C18 will be unsatisfied.

Fig. S3b shows the function p_n in TMB as $\mathscr{M}_n$ and $\mathscr{B}_n$ varies for a tuned c_m and c_b. We can observe that probability of flipping a node increases when $\mathscr{M}_n$ increases and $\mathscr{B}_n$ decreases. In other words, the system stochastically flips variables that are are involved in more unsatisfied clauses and fewer *break* clauses.

Relation between $\mathscr{M}_n$, $\mathscr{B}_n$ and the dynamics of cBRIM

Let us consider the Hamiltonian (H_n) of only those clauses, $C_i = (\ell_{i1} \vee \ell_{i2} \vee \ell_{i3})$ involving variable x_n:

$$H_n = \sum_{i|x_n \in C_i} g(\ell_{i1})g(\ell_{i2})g(\ell_{i3})$$
$$\text{where, } g(\ell_{ij}) = \begin{cases} (1-x_a) & \ell_{ij} = x_a \\ x_a & \ell_{ij} = \neg x_a \end{cases} \text{ and } x_a \in \{0,1\} \tag{1}$$

Without loss of generality, let us consider literal ℓ_{i1} to be associated with variable x_n. After expanding H_n, for each clause C_i, we focus only on the terms containing x_n, referred to as $\mathscr{T}_{i|x_n}$.

$$\mathscr{T}_{i|x_n} = \begin{cases} -g(\ell_{i2})g(\ell_{i3}) & \ell_{i1} = x_n \\ +g(\ell_{i2})g(\ell_{i3}) & \ell_{i1} = \neg x_n \end{cases} \tag{2}$$

We define C_i to be x_n-UNSAT if and only if subclause $(\ell_{i2} \vee \ell_{i3})$ is *unsatisfied*. Suppose that C_i contains x_n. Using the definition of $g(\ell_{ij})$ in Eq. 1, if C_i is x_n-UNSAT, then $\mathscr{T}_{i|x_n} = -1$ and 0 otherwise. On the contrary, if C_i contains the negated literal $\neg x_n$, then $\mathscr{T}_{i|x_n} = 1$ in the case that C_i is x_n-UNSAT.

We now connect these definitions with the concepts of $\mathscr{M}_n$ and $\mathscr{B}_n$. A key observation is that C_i contributes to $\mathscr{M}_n$ or $\mathscr{B}_n$ if and only if it is x_n-UNSAT. If $x_n = 1$, then all clauses with $\mathscr{T}_{i|x_n} = 1$ has $\ell_{i1} = \neg x_n = 0$. Thus, such clauses will contribute to $\mathscr{M}_n$. While all clauses with $\mathscr{T}_{i|x_n} = -1$ contribute to $\mathscr{B}_n$. Similarly, if $x_n = 0$, then all clauses with $\mathscr{T}_{i|x_n} = -1$ are counted in $\mathscr{M}_n$, and all clauses with $\mathscr{T}_{i|x_n} = 1$ are counted in $\mathscr{B}_n$. To summarize:

$$\sum_{i|x_n \in C_i} \mathscr{T}_{i|x_n} = (1 - 2x_n) \times (\mathscr{B}_n - \mathscr{M}_n) = \begin{cases} \mathscr{M}_n - \mathscr{B}_n & x_n = 1 \\ \mathscr{B}_n - \mathscr{M}_n & x_n = 0 \end{cases} \tag{3}$$

Moreover, $\mathscr{T}_{i|x_n}$ is only derived from terms containing x_n. This implies, $\frac{\partial H}{\partial v_n} = \sum_{i|x_n \in C_i} \mathscr{T}_{i|x_n}$. Therefore, the differential equation for AIMS becomes:

$$\frac{dv_n}{dt} = -\alpha \frac{\partial H}{\partial v_n} = -\alpha \sum_{i|x_n \in C_i} \mathscr{T}_{i|x_n} = \alpha(1 - 2x_n) \times (\mathscr{M}_n - \mathscr{B}_n) \tag{4}$$

Hardware implementation of TMB

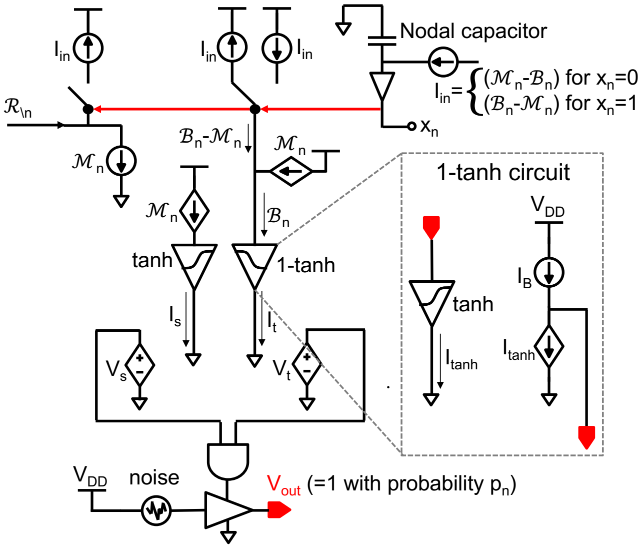

Supplementary Figure S4. The CMOS circuit implementation of the heuristic.

With $\mathscr{M}_n$ and $\mathscr{B}_n$ represented as currents, we can design a circuit to compute p_n for TMB with approximate tanh generators and using an AND gate to approximate the multiplication. Fig. S4 shows an example implementation. The current $\mathscr{R}_{\backslash n}$ is generated using coupling units not shown in the figure. Based on the quantized state of the node x_n, a switch is set to ensure the current flowing into the $(1 - \tanh)$ circuit is always $\mathscr{B}_n$ and also to get $\mathscr{M}_n$ from $\mathscr{R}_{\backslash n}$. The tanh function is implemented by modifying a differential current-mode implementation shown in a recent work[1]. The resulting circuit is shown in Fig. S5. Using this, $(1 - \tanh)$ is trivial to implement as shown in the zoomed-in part of Fig. S4. The parameters c_m and c_b are tuned by applying a gain to the current-controlled current source that mirrors the input current in the tanh circuit. The output currents of the tanh and $(1 - \tanh)$ circuits, referred to as I_s and I_t, are used to control the current-controlled voltage sources V_s and V_t respectively. Note that, $I_s, I_t \in [0, I_{bias}]$ because the input currents, $\mathscr{M}_n$ and $\mathscr{B}_n$ are positive. V_s and V_t produce voltages in the range $[0, V_{DD}]$ which are fed as inputs to an AND gate to multiply approximately. The output of AND gate controls the

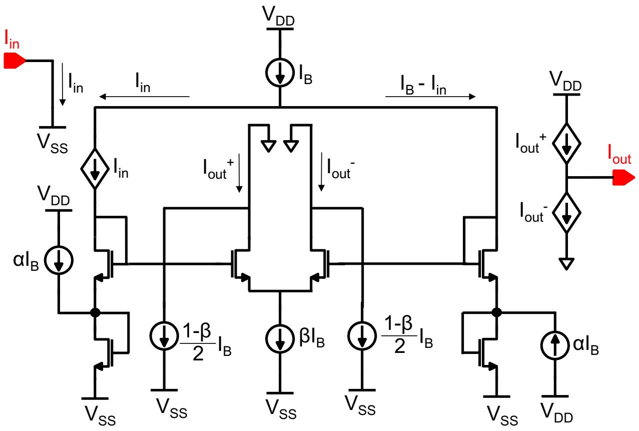

Supplementary Figure S5. CMOS implementation of a single-ended tanh circuit.

probability p_n of a stochastic circuit that outputs $V_{out} = 1$ with a probability p_n. This way, each node can be selected by our TMB heuristic to flip if $V_{out} = 1$. With this circuit, we measure a delay of 1 ns in Cadence simulation to select a node to flip.

While the TMB heuristic requires extra hardware, it is only needed per node and thus comes with $O(N)$ cost. The system's overall circuit complexity is dominated by that of the $O(N^2)$ couplers.

Generating $\mathscr{M}_n$

Let us consider the Hamiltonian (H_n) of only those clauses (C_i) involving node n (variable x_n). This function has some terms that contain v_n (we call them collectively $\mathscr{R}_n$) and the remaining that do not ($\mathscr{R}_{\setminus n}$).

$$\begin{aligned} H_n &= \sum_{i|x_n \in C_i} g(\ell_{i1})g(\ell_{i2})g(\ell_{i3}) \\ &= const. - l_n v_n - \sum_j q_{nj} v_n v_j - \sum_{jk} c_{njk} v_n v_j v_k \\ &= \mathscr{R}_n + \mathscr{R}_{\setminus n} = \mathscr{M}_n \end{aligned} \tag{5}$$

A simple but key insight of the nature of H_n is that, a clause C_i is unsatisfied if and only if the product $g(\ell_{i1})g(\ell_{i2})g(\ell_{i3}) = 1$. Otherwise, C_i is satisfied with the product being 0. Therefore, $H_n = \mathscr{M}_n$. We know that, the incoming current of each node is simply $\frac{dv_n}{dt} = -\alpha \frac{\partial \mathscr{R}_n}{\partial v_n}$. This is because only $\mathscr{R}_n$ contains terms with v_n. Using this fact in Eq. 5,

$$\begin{aligned} \mathscr{M}_n &= x_n \frac{\partial \mathscr{R}_n}{\partial v_n} + \mathscr{R}_{\setminus n} \\ &= -\frac{x_n}{\alpha} \cdot \frac{dv_n}{dt} + \mathscr{R}_{\setminus n} \\ &= x_n(\mathscr{M}_n - \mathscr{B}_n) + \mathscr{R}_{\setminus n} \end{aligned} \tag{6}$$

where x_n is the quantized state of the node. While deriving Eq. 6, we use a simplification that since $x_n \in \{0, 1\}$, we can write $x_n^2 = x_n$. The first term in Eq. 6 can be obtained from the incoming current and only contributes when $x_n = 1$. The second term ($\mathscr{R}_{\setminus n}$) can be computed by generating an electric current just like the regular coupling units. As a result, we generate a current proportional to $\mathscr{M}_n$.

Impact of clamp time on AIMS

In a fast dynamical system like AIMS, stochastically flipping a node with TMB heuristic is achieved by *clamping* the node to a fixed polarity using a large current driver for a specific duration. Assuming the system started in a fixed point (local minimum), too short and too few of these clampings will almost certainly result in the voltages reverting back quickly to the prior value.

By its very nature, our design flips nodes in an asynchronous manner and allows multiple stochastically overlapping flips. Lengthening the duration increases the number of overlaps. Fig. S6a shows the success probability of AIMS while solving 20 different 500-variable problems with constant annealing time of 0.44 ms and increasing node clamp time. From the figure, we can observe that too short clamp times result in poor solution quality. In this case, we conjecture that the system may not get

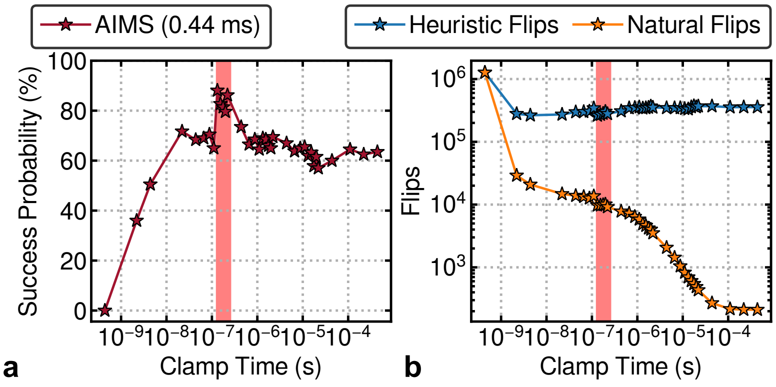

Supplementary Figure S6. Analysis of AIMS with respect to clamp times. a) Success probability (%) of AIMS while solving 20 instances of 500-variable problems with a cutoff time of 0.44 ms and increasing node clamp time. b) The number of heuristic and natural flips as node clamp time increases. The vertical red region shows the optimal clamp times.

enough time to react to the flips. There are some optimal clamp times (shown by the "red" vertical region) resulting in the best success probability. Fig. S6b shows the number of flips done by our TMB heuristic and the "natural" flips done solely by the dynamical system. We notice that the amount of natural flips is very high for very short clamp time that results in poor solution quality. This number reduces as clamp time increases, with the optimal region having roughly 10^4 natural flips. The heuristic flips increases slightly after a rapid drop initially and are much higher than natural flips.

Impact of ground state latching on performance

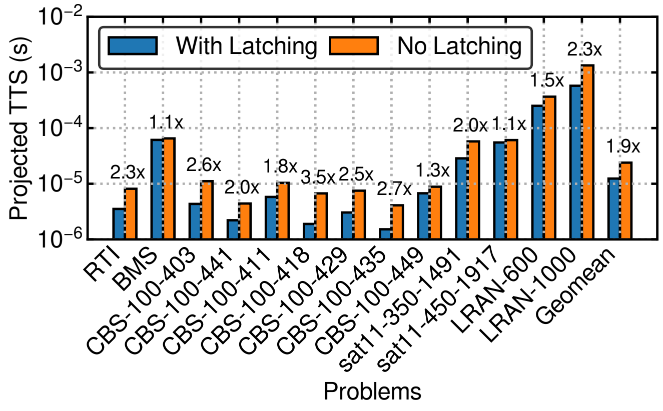

Supplementary Figure S7. TTS of AIMS with and without ground state latching.

We also identify another optimization thanks to the TMB heuristic. To understand this, recall that the formulation of the SAT Hamiltonian in is very convenient to check for a satisfying solution ($H = 0$). This is also equivalent to checking if $\forall n(\mathscr{M}_n = 0)$. The TMB hardware in AIMS already provides $\mathscr{M}_n$ in the form of an electric current. Hence, we add support to check the condition and terminate the annealing when it is satisfied, referred to as *latching*. Fig. S7 shows the best TTS AIMS can obtain with and without latching. In general, we find that with this logic, TTS improves by 1.9x compared to the best TTS obtained without latching. We note that beyond this speed improvement, the latch logic is likely to be of important *practical* value. It reduces the reliance on the user to set a judicious annealing time.

Sensitivity to variation in parameters and devices

In this section, we look at how variation in parameters and devices can impact the performance of AIMS. Fig. S8a and Fig. S8b shows the heatmap of TTS as parameters, c_m and c_b varies while solving uniform random and scale-free problems respectively with 500 variables. We can observe that both the heatmaps have an optimal region (see darker regions towards the middle) for the parameter values resulting in the least TTS. While the range of optimal c_m is largely the same, the optimal c_b values seem to vary slightly for both the problem types: $[0.48, 0.63]$ vs $[0.35, 0.41]$. Another important observation is that very low c_m and c_b

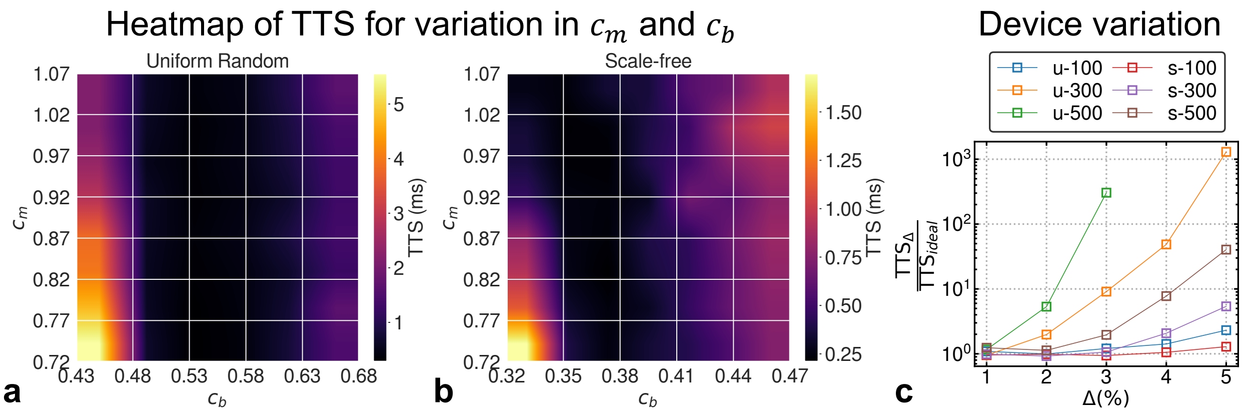

Supplementary Figure S8. Impact of variation in parameters and devices. Heatmap of TTS for AIMS due to variation in parameters, c_m and c_b for a) uniform random and b) scale-free problems with 500 variables. The heatmap is an interpolation of the geometric mean TTS of 20 different problems run for several different values of c_m and c_b. c) The ratio of TTS with (TTS_Δ) and without device variation (TTS_{ideal}) as percentage of variation, $\Delta(\%)$ increases for both uniform random (u-N) and scale-free (s-N) problems, where number of variables, $N \in \{100, 300, 500\}$.

severely increases the TTS as shown by the yellow region towards the bottom left. Thus, small variation in parameters doesn't impact the performance significantly.

Next, we introduce device variation into our simulation of AIMS as follows:

1. Each tanh circuit in our implementation of TMB, incorporates device mismatch and process variation obtained from Cadence by running a random Monte Carlo sampling in the Virtuoso Analog Design Environment XL. This sampling applies device mismatch and process variation, and is performed on a DC analysis by sweeping the input and measuring the output to obtain a transfer function. 1000 samples are taken for each circuit block, yielding 1000 curves relating the output to the input. These are then randomly sampled on a device-by-device basis in the behavioral model to estimate the impact on performance.

2. Each nodal capacitor and coupling resistor has zero mean additive Gaussian noise with standard deviation, Δ.

Fig. S8c shows a plot of the ratio of TTS with and without variation $\left(\frac{TTS_\Delta}{TTS_{ideal}}\right)$ as we increase the percentage of device variation from the ideal value, $\Delta(\%)$. Each plot is obtained by calculating the geometric mean TTS for 50 different problems and the same annealing times as the ideal runs. We can make some key observations. First, the industrial-like scale-free problems (s-100, s-300 and s-500) scale much better than their uniform random counterparts (u-100, u-300 and u-500). While further research is required to explain such behaviour, we note here that industrial instances mostly follow scale-free distributions[2–4]. Second, for a given variation, the larger the problem size, the more degradation in TTS. While a deviation of 3-4% can be tolerated, larger deviations might be detrimental especially for bigger problem sizes, which demand mitigation techniques.

Hardware parameters

Supplementary Table S1. Comparison of hardware parameters of various Ising machines.

	ISSCC 2020[5]	ISSCC 2021[6]	Nature Elec. 2022[7]	ISSCL 2023[8]	This work
**# of spins**	512	$16K$	4793	128 – 1024	512
**High-degree interactions**	No	No	No	Yes	Yes
**Hardware topology**	Complete graph	King's Graph	Sparse Graph	King's Graph	Complete Graph
**Target problem type**	MaxCut	MaxCut	Factorization and 3-SAT	3-SAT	3-SAT
**Chip area**	$12\,mm^2$	$10.812\,mm^2$	NA	$0.345\,mm^2$	$169\,mm^2$
**Power**	$649\,mW$[1]	NA	NA	$477.6\,\mu W$	$300\,mW$
**Speedup over SAT solvers**	NA	NA	No	No	Yes (100 to 1000×)

[1]Reported peak power.

Table S1 shows the hardware parameters of existing Ising machines compared to this work. Some key points to note: 1) Majority of Ising machines do not support high-degree interactions. 2) Most Ising machines only support sparse hardware topology thus limiting its usability. 3) Although our proposed Ising machine takes more chip area, to the best of our knowledge, it is the only one projected to outperform state-of-the-art SAT solvers by orders of magnitude.

The reported power consumption for the ISSCC 2020 work is the measured peak power while solving an all-to-all connected MaxCut graph. In contrast, AIMS targets 3-SAT problems which are very sparse ($\sim$2% density). This implies that majority (98%) of the coupling units in AIMS are switched off leading to much lower power consumption.

References

1. Carrasco-Robles, M. & Serrano, L. A novel cmos current mode fully differential tanh (x) implementation. In *2008 IEEE International Symposium on Circuits and Systems (ISCAS)*, 2158–2161, DOI: 10.1109/ISCAS.2008.4541878 (2008).

2. Ansótegui, C., Bonet, M. L. & Levy, J. Towards industrial-like random sat instances. In *Proceedings of the 21st International Joint Conference on Artificial Intelligence*, IJCAI'09, 387–392 (Morgan Kaufmann Publishers Inc., San Francisco, CA, USA, 2009).

3. Ansótegui, C., Bonet, M. L. & Levy, J. On the structure of industrial SAT instances. In *Principles and Practice of Constraint Programming-CP 2009: 15th International Conference, CP 2009 Lisbon, Portugal, September 20-24, 2009 Proceedings 15*, 127–141 (Springer, 2009).

4. Friedrich, T., Krohmer, A., Rothenberger, R. & Sutton, A. Phase transitions for scale-free SAT formulas. In *Proceedings of the AAAI Conference on Artificial Intelligence*, vol. 31 (2017).

5. Yamamoto, K. *et al.* Statica: A 512-spin 0.25m-weight annealing processor with an all-spin-updates-at-once architecture for combinatorial optimization with complete spin–spin interactions. *IEEE J. Solid-State Circuits* **56**, 165–178, DOI: 10.1109/JSSC.2020.3027702 (2021).

6. Takemoto, T. *et al.* 4.6 a 144kb annealing system composed of 9×16kb annealing processor chips with scalable chip-to-chip connections for large-scale combinatorial optimization problems. In *2021 IEEE International Solid- State Circuits Conference (ISSCC)*, vol. 64, 64–66, DOI: 10.1109/ISSCC42613.2021.9365748 (2021).

7. Aadit, N. A. *et al.* Massively parallel probabilistic computing with sparse ising machines. *Nat. Electron.* **5**, 460–468, DOI: 10.1038/s41928-022-00774-2 (2022).

8. Su, Y., Kim, T. T.-H. & Kim, B. A reconfigurable cmos ising machine with three-body spin interactions for solving boolean satisfiability with direct mapping. *IEEE Solid-State Circuits Lett.* **6**, 221–224, DOI: 10.1109/LSSC.2023.3303332 (2023).
